# The great catastrophe: causes of the Permo-Triassic marine mass extinction

**DOI:** 10.1093/nsr/nwad273

**Published:** 2023-10-25

**Authors:** Paul B Wignall, David P G Bond

**Affiliations:** School of Earth & Environment, University of Leeds, Leeds LS2 9JT, UK; School of Environmental Sciences, University of Hull, Hull HU6 7RX, UK

**Keywords:** ocean anoxia, ocean acidification, siltation, productivity collapse, volcanic winter

## Abstract

The marine losses during the Permo-Triassic mass extinction were the worst ever experienced. All groups were badly affected, especially amongst the benthos (e.g. brachiopods, corals, bryozoans, foraminifers, ostracods). Planktonic populations underwent a fundamental change with eukaryotic algae being replaced by nitrogen-fixing bacteria, green-sulphur bacteria, sulphate-reducing bacteria and prasinophytes. Detailed studies of boundary sections, especially those in South China, have resolved the crisis to a ∼55 kyr interval straddling the Permo-Triassic boundary. Many of the losses occur at the beginning and end of this interval painting a picture of a two-phase extinction. Improved knowledge of the extinction has been supported by numerous geochemical studies that allow diverse proposed extinction mechanisms to be studied. A transition from oxygenated to anoxic-euxinic conditions is seen in most sections globally, although the intensity and timing shows regional variability. Decreased ocean ventilation coincides with rapidly rising temperatures and many extinction scenarios attribute the losses to both anoxia and high temperatures. Other kill mechanisms include ocean acidification for which there is conflicting support from geochemical proxies and, even less likely, siltation (burial under a massive influx of terrigenous sediment) which lacks substantive sedimentological evidence. The ultimate driver of the catastrophic changes at the end of the Permian was likely Siberian Trap eruptions and their associated carbon dioxide emissions with consequences such as warming, ocean stagnation and acidification. Volcanic winter episodes stemming from Siberian volcanism have also been linked to the crisis, but the short-term nature of these episodes (<decades) and the overwhelming evidence for rapid warming during the crisis makes this an unlikely cause. Finally, whilst the extinction is well studied in equatorial latitudes, a different history is found in northern Boreal latitudes including an earlier crisis which merits further study in order to fully understand the course and cause of the Permo-Triassic extinctions.

## Teaser

The mass extinction at the end of the Permian was the most severe in Earth’s history. Its causes are debated with marine anoxia, global warming and ocean acidification amongst popular kill mechanisms but the quality of the supporting evidence varies.

## INTRODUCTION

The mass extinction at the end of the Permian, nearly 252 million years ago, was the most severe to affect life on Earth. Generic extinction rates exceeded 80%, with victims encountered in all environments from the open oceans to polar forests [1–3]. The cause and pace of this catastrophe has been long debated, but the contemporaneous eruption of the giant Siberian Traps flood basalt province, first recognised over 30 years ago, has focussed attention on the effects of this giant ‘smoking gun’. Meteorite impact is famously implicated in the Cretaceous-Palaeogene mass extinction but attempts to find evidence for an end-Permian impact have not proved convincing [4] leaving volcanism as the principal contender. Volcanism releases a diverse range of volatiles that have potentially damaging effects on climate and life, most notably sulphur- and carbon dioxide along with halogens and water vapour. Most extinction mechanisms focus on these emissions and many scenarios involve the long-lasting effects of CO_2_ and its cascade of consequences (warming, ocean stagnation, ocean acidification, siltation). For clarity we examine each cause separately, because each has potentially different consequences for marine life, and they need not be equally harmful. In reality there are many synergistic interactions between these stresses (e.g. high temperatures can cause direct stress through protein denaturing but they also cause increased oxygen demand, and yet warmer waters hold less dissolved oxygen; a rather unfortunate combination [5]). But, before examining the evidence for some of the proposed causes of marine extinctions (terrestrial extinction mechanisms are not considered here), we review the current understanding of the timing of Permo-Triassic marine biodiversity losses. It is important, when attempting to attribute cause-and-effect in deep time, to show that the temporal relationships are correct. Indeed, it is one of the principal tests available to historians of the ancient world.

**Figure 1.**
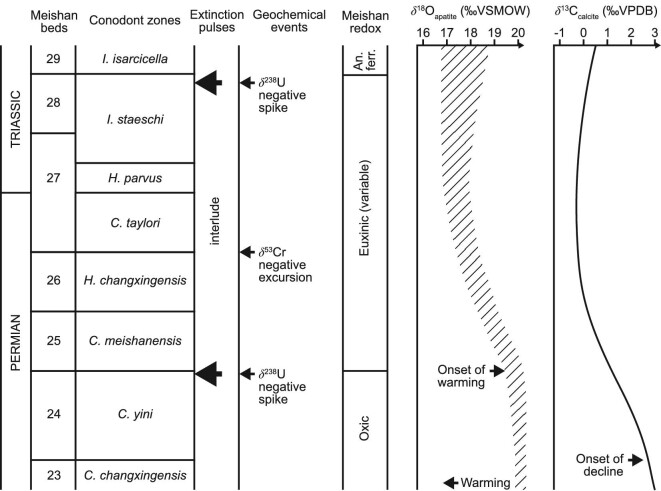
Summary of geochemical, environmental and faunal events across the Permo-Triassic boundary. The conodont zonation scheme is compared with the bed numbering scheme of the Meishan global stratotype in South China [6]. The global trend in carbonate isotope values [3] and evidence for major redox changes, surface water temperature changes (based on oxygen isotope ratios obtained from conodonts) is discussed in the main text. an. ferr. = anoxic ferruginous. Conodont genera are *I. = Isarcicella, H. = Hindeodus* and *C. = Clarkina*.

Intensive research over the past few decades has ensured that the best-known record of the Permo-Triassic marine mass extinction (PTMME) is from South China [3,6–9]. Strata in the region were deposited in warm, equatorial seas in a broad range of environments ranging from shallow-water platform carbonates to deep-water basinal cherts and mudrocks. These have allowed the crisis to be examined over a range of palaeo-water depths. In addition, intensive study of the rapidly evolving conodonts, a group of tiny, eel-like vertebrates with phosphatic teeth, has allowed temporal and spatial losses to be resolved at a resolution that would have been extraordinary only a few decades ago: some conodont zones are probably only a few tens of thousands of years in duration. No section has been studied in greater detail than that at Meishan, a disused quarry in Zheijiang Province, where a moderately deep-water record of limestones and calcareous mudstones has been designated the Global Stratotype Section and Point (GSSP) for the Permo-Triassic boundary [6]. Bed numbers at this location provide an alternative reference frame for the PTMME that are often used in combination with the conodont zonation scheme (Fig. 1).

At Meishan, and elsewhere in South China, the major phase of the PTMME occurs in the latest Permian at the boundary between the *Clarkina yini* and *C. meishanensis* zones [9,10]. This level is at the top of Bed 24 at Meishan, a bioclastic limestone that is overlain by a thin tuff (Bed 25) (Fig. 1). The transition marks the loss of more than 200 species across the region, an extinction magnitude of ∼60%, that may have occurred in as little as 1000 years [11]. Losses were especially severe amongst some shallow-water groups such as calcareous algae, fusulinid foraminifers, gastropods and ammonoids, whilst ostracods and conodonts were little affected at this level [7,12,13]. This crisis has long been known as the end-Permian mass extinction and many earlier studies (and even some recent ones [8,14]) considered this phase to mark the end of a prolonged period of diversity decline. In contrast, this crisis is more widely regarded as being geologically rapid, with losses occurring within a few thousand years [11,15,16]. The subsequent interval, straddling the Permo-Triassic boundary, is intriguing in that the marine fauna consists of a mixture of Permian survivors and newly originating species [9]. In South China, and elsewhere in equatorial latitudes, this level also sees the proliferation of microbialities and oolites in shallow-water carbonate sections [17]. The majority of this ‘mixed fauna’ is lost during a second, equally rapid pulse of enhanced extinction rates in the earliest Triassic *Isarcica staeschi* Zone, around the top of the Bed 28 boundary at Meishan [7,10,13]. Species-level losses reach 70% at this level, with many ostracods, brachiopods and small foraminifers disappearing. The mass extinction is thus a two-step event that straddles the Permo-Triassic boundary, with an interlude between crises of ∼55 kyr in duration [3].

**Figure 2.**
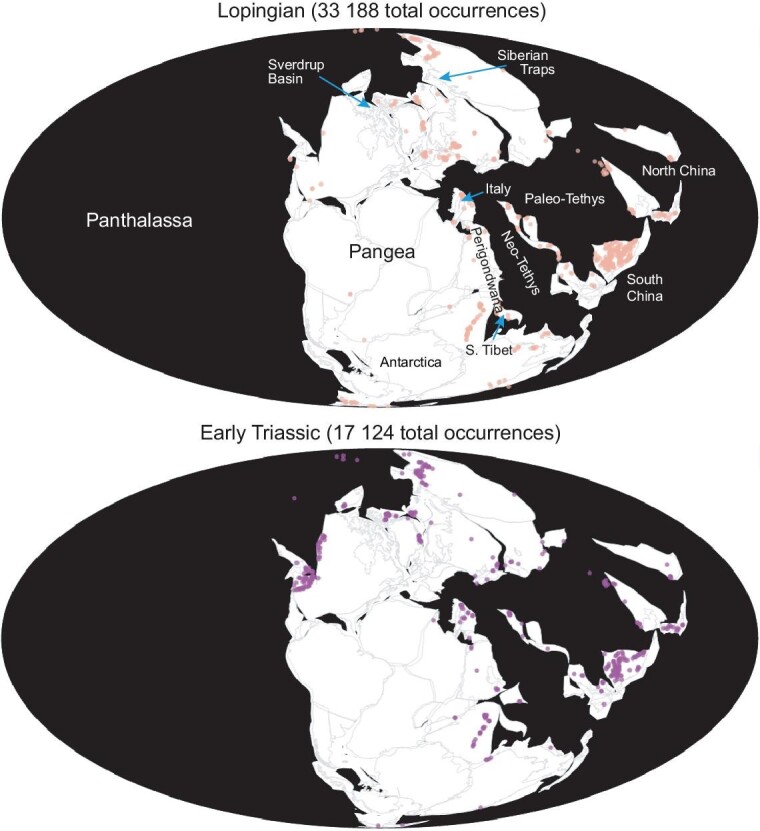
Lopingian and Early Triassic palaeogeographic maps showing the occurrences of common marine groups (ammonoids, bivalves, brachiopods, conodonts, corals (rugose and tabulate), foraminifers, ostracods). Data and maps from the Paleobiology Database (accessed 12th July 2023). As shown by the distribution of data, much of our knowledge of the PTME derives from South China. Key geographical areas mentioned in the text are labelled on the Lopingian map.

**Figure 3.**
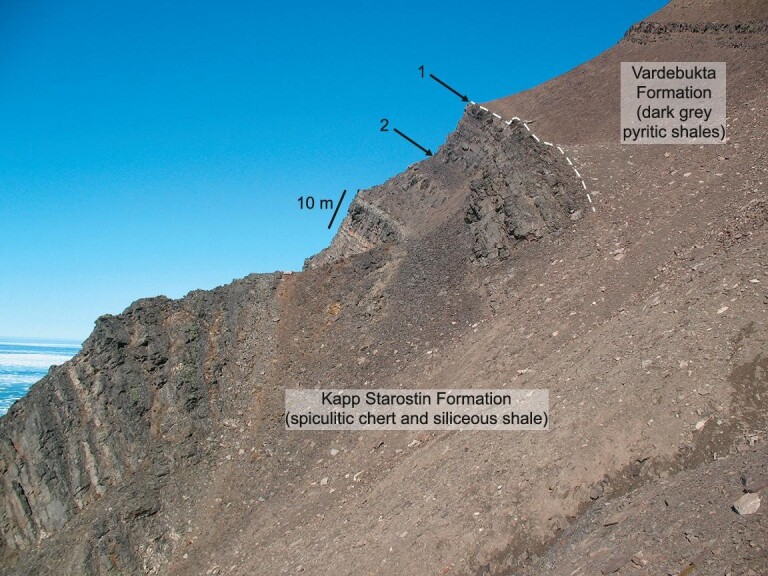
Field photo of the Permian-Triassic transition at Bravaisberget cliff section in southern Spitsbergen showing levels of extinction losses. Siliceous sponges disappear at level 1 and is marked by the loss of cherts at the top of the Kapp Starostin Formation. The Permo-Triassic boundary occurs a few metres above this horizon. However, most extinctions in this section occur at a lower point, labelled 2, when a diverse shelly benthos, dominated by brachiopods and bryozoans, disappears. This is estimated to be ∼2.33 myrs before the level 1 extinction.

Our knowledge of the PTMME in other regions of the world lags behind that of South China, which is by far the best studied region. For example, very little is known of the extinction losses in the southern hemisphere, other than mid-latitude locations on the Perigondwanan margin (Fig. 2). Nonetheless, the available evidence suggests a similar crisis history in other areas. Deep-water records from western Canada indicate a double-pulse extinction, with radiolarians disappearing before siliceous sponges, whilst condensed carbonates from the South Tibetan microcontinent, located in moderately high southern palaeolatitudes, also record two crises straddling the Permo-Triassic boundary [18]. The shallow-water carbonates of the Dolomites, northern Italy, similarly show two extinction levels: the first occurs at the base of an oolite-bearing bed (the Tesero Oolite Horizon) that eliminated small foraminifers and brachiopods, whilst the second, at the top of the bed, removed calcareous algae and fusulinid foraminifers [19]. The second extinction pulse occurs above the first appearance of *Hindeodus parvus*, the conodont that defines the base of the Triassic, and is thus approximately at the same level as the second pulse in South China. However, not all regions record this two-pulse pattern. The shelf seas of Spitsbergen in the Svalbard archipelago, located in northern Boreal latitudes, saw diverse shelly groups (notably brachiopods, bivalves, bryozoans, foraminifers) disappear considerably below the Permo-Triassic boundary although siliceous sponges and a large, soft-bodied burrowing fauna persisted to a level shortly above the onset of a negative δ^13^C_org_ excursion [20]. The first phase of extinction of the PTMME happens at the same level relative to the C isotope record in lower latitudes indicating that the Spitsbergen sponge extinction was likely contemporary, however the losses of shelly groups must have been considerably earlier. Biostratigraphic indicators are lacking from the Upper Permian strata in Spitsbergen, but the available chemostratigraphy from the key Festningen section places the base of the Late Permian 40 m below the Permo-Triassic boundary [21] whilst the shelly taxa are lost 12 m below the boundary. Taking a duration of 7.65 million years for the Late Permian [22], and assuming constant sedimentation rates (spiculitic cherts dominate the Spitsbergen section at this level [21]), indicates the shelly benthos went extinct 2.3 million years below the boundary. This is a level at the base of the topmost Permian Changhsingian Stage, considerably earlier than the onset of the PTMME (Fig. 3).

## CAUSES OF THE PERMO-TRIASSIC MARINE MASS EXTINCTION

### Anoxia

The concept of anoxia-driven PTMME is supported by an abundance of sedimentological, palaeoecological, petrographic and geochemical studies [2,23–25]. These broadly indicate the importance of anoxia at this time although different proxies respond to different intensities of oxygen depletion and some inform about local rather than global conditions. Finely laminated, pyritic sediments record local conditions but their widespread occurrence in Permo-Triassic boundary sections in settings from shallow shelf seas to the abyssal plain indicates the extent of global anoxia (Fig. 3). Note that abyssal facies are found in accreted oceanic terranes [15,26–28]. Pyrite framboid populations provide clues to the intensity and variability of this anoxia. Framboids form in weakly reducing conditions and their size range provides a useful method of distinguishing a range of oxygen-restricted conditions from oxygen-poor (dysoxic) bottom waters to sulfidic (euxinic) waters [29]. Analysis of framboid populations in samples from diverse locations shows that the PTMME coincides with the onset of oxygen-poor conditions, albeit with considerable regional variations: high latitude Boreal sections experienced generally more persistent and intensely anoxic/euxinic conditions than lower latitude Tethyan locations [29]. Even the abyssal depths of the Panthalassa Ocean record euxinia [26,28,30]—an extraordinary occurrence because the ocean depths were generally well ventilated after the Early Palaeozoic thanks to vigorous circulation [31].

The ocean's uranium isotope ratios are recorded in the archive of limestones and dolostones. The values reflect the extent of global anoxia because of the preferential removal of U-238 in anoxic sediments, which leaves seawater isotopically lighter with respect to uranium isotopes [32]. Thus, the discovery of two negative δ^238^ U excursions at the level of the two mass extinction phases in a western Tethyan dolomite record provides strong evidence of intense drawdown of seawater uranium suggesting that expansion of marine anoxia was responsible for both crises [33]. Other proxies for global anoxia broadly support the anoxia-extinction link although they differ in their timing. The chromium isotope record (δ^53^Cr) shows an abrupt switch to lighter values at the base of the *C. taylori* zone in South China, which can be attributed to quantitative reduction and precipitation of Cr (VI) due to the expansion of ocean anoxia [34]. Curiously, the timing of this transition places the onset of anoxia between the two pulses of mass extinction. Thallium isotopes provide a novel proxy of oceanic redox that reflects the global extent of the element's drawdown in manganese oxide deposits [35]. Data from condensed Panthalassan sites show a gradually increasing ϵ^205^Tl trend from the Middle Permian and into the Triassic which is attributed to the gradual decline of Mn oxide burial as the oceans deoxygenated. However, a brief negative excursion in the latest Permian is attributed to re-invigorated thermohaline circulation during a cold interval [35]. The problem with this scenario is that the negative ϵ^205^Tl excursion occurs at different stratigraphic levels in all three study sections, with two occurring before the mass extinction. Furthermore, the long-term trend of increasing ocean anoxia in the Late Permian, suggested by the ϵ^205^Tl trends, is not supported by other geochemical proxies. Redox-sensitive trace metal concentrations and iron speciation data from deep-water Panthalassa sections all indicate that anoxia only developed at the onset of the carbon isotope excursion and thus shortly before the onset of the crisis [28,32,36].

The intensity and extent of anoxia during the PTMME was sufficient to deplete the ocean of its redox-sensitive trace metals [37,38]. Thus, the trace metal enrichment that is typically recorded in euxinic strata is not seen in some sections [38], although the slowly accumulating euxinic sediments on the Panthalassa abyssal plain do have enhanced trace metal concentrations [28,39]. This drawdown occurs in a predictable pattern, with U depletion occurring slightly before the mass extinction, suggesting increased burial in anoxic ocean sediment [32], but it was not until after the first phase of mass extinction that euxinia became sufficiently widespread for metals such as vanadium and molybdenum to become depleted [38,39]. The decline in the oceanic trace metal inventory probably accounts for claims of improved levels of oxygenation at some sites during the PTMME [40]. For example, the Shangsi section in the Upper Yangtze Basin has a trace metal signature characteristic of oxygenated conditions during the mass extinction interval, which has led to the suggestion that anoxia was not responsible [40]. But this conclusion is not supported by iron speciation data from the same levels which show an increase in the ratio of pyrite iron to reactive iron to 0.4 at this time. This value is indicative of anoxic ferruginous conditions [41]. Pyrite framboids do not form under such conditions, and no framboids are found in the extinction interval at Shangsi (cf [29]) but the ferruginous anoxic conditions were hostile enough to cause the shutdown of bioturbation and the onset of finely laminated sedimentation [42].

Intriguingly, redox studies of the Meishan section have provided slightly different oxygenation histories [43]. Iron speciation and trace metal trends indicate that the onset of intermittently euxinic conditions began at the top of Bed 24 and persisted up to Bed 28 when ferruginous anoxic conditions were developed [38]. Organic biomarkers for photic zone euxinia are common in Bed 24 and Bed 27 but they occur in reduced abundance in between [44]. In contrast, pyrite framboids are either rare in Bed 24 [45] or abundant and small [46] depending on which study is consulted. The strata between the two extinction horizons (tops of beds 24 and 27) are well bioturbated [27] and either lack framboids [45] or have larger sized populations suggestive of dysoxic conditions [46]. Instead, marcasite is the most abundant iron sulphide phase in this level [47], the significance of which is discussed below. The reason for the discrepancy between framboid studies may lie in the intrinsically rapid fluctuations of redox conditions recorded in the Permo-Triassic boundary strata ensuring that even closely spaced samples may record different conditions. Overall, the two extinction levels at Meishan show reasonable correlation with intensely anoxic episodes, with the intervening interval being marked by the mixed fauna and highly unstable redox conditions that at some times saw the development of photic zone euxinia whilst at others supported a bioturbating infauna.

The extent and intensity of anoxia in the world's oceans during the PTMME was unparalleled in the Phanerozoic and there have been many attempts to investigate the conditions responsible for this using ocean-climate models. Most initial attempts were unsuccessful [2], but more recently modelling has successively replicated many observed phenomena. Using the Community Earth System Model (CESM), Penn *et al.* [23] showed that a rapid increase of atmospheric CO_2_ at the end of the Permian would have driven ocean warming and stagnation due to increased freshwater runoff into the northern Boreal Ocean, triggered by increased rainfall at high latitudes. The resultant density stratification weakened deep-water formation thus causing poorly-circulated global oceans. The CESM output has the most intense anoxia occurring in high latitude settings whilst, in contrast, low latitudes, especially in western Tethys and along the Perigondwanan margin, experienced less intense oxygen depletion due to a decline in nutrient supply and weakened microbial oxygen demand in these regions [23]. These model outputs are in good agreement with the evidence from the field: anoxia was most weakly developed in Perigondwana [18,27], whilst Boreal anoxia was especially intense even in nearshore settings [29]. It is notable that claims that anoxia did not feature in the PTMME tend to be based on the evidence for weak oxygen restriction in western Tethyan sections [19]. Another recent modelling effort, using the cGEnIE earth-system model, produces anoxia by a temperature-driven increase of microbial respiration rates [48]. The resultant increased oxygen consumption causes mid-water oxygen-minimum zones to expand downwards, into deeper waters, and upwards to flood shelf seas with anoxic waters. However, the model fails to replicate the observed regional variations in the intensity of anoxia (western Tethys and Perigondwana become more deoxygenated than eastern Tethys in the cGEnIE model, the opposite to what is observed), suggesting that, whilst important, respiration rate changes were not a key factor in the development of anoxia.

### Warming

In most extinction scenarios, warming is seen as a key component in driving ocean anoxia and the associated loss of marine habitat at the end of the Permian [23,27,49,50]. There is considerable palaeontological evidence to support this notion. In South China the first phase of extinction saw many surviving foraminifers migrate to deeper waters, a trend that is reasonably interpreted as a response to avoid lethally hot surface waters [13]. Similarly, some equatorial taxa undertook long-distance migration to the cooler water of high latitude Perigondwanan locations during the Permo-Triassic transition [18,51]. These refugee populations survived into the earliest Triassic but succumbed to the second extinction phase. Considering other groups, the selective loss of high-temperature intolerant groups such as corals and (nearly all) radiolarians in the first extinction pulse again suggests high temperatures were the main driver of extinction amongst surface-dwelling groups (although see further discussion of radiolarian losses below, many of which slightly predate the onset of warming) [50]. Dysoxic-to-anoxic conditions then played the leading role in the second extinction pulse, especially for deeper-water benthos [50]. More broadly, analysis of global palaeontological databases has provided equivocal evidence for the role of temperature-driven extinction. Penn *et al.* [23] identified a substantially higher generic extinction rate for marine genera in northern high latitudes compared with those in the tropics, suggesting a potential temperature-control, whilst in contrast Reddin *et al.* [52] suggested there was only weak evidence for selective losses at temperate latitudes. Looking at the global data in greater detail, the northern high latitude losses are concentrated amongst benthic groups suggesting that cool-adapted, bottom-living taxa did not fare well [53] (although the more intense anoxia in these latitudes—see above—could also be a factor in these benthic losses).

The extreme warmth of the post-extinction Early Triassic world is amply demonstrated by a wealth of terrestrial sedimentary data [54,55] and marine δ^18^O_phosphate_ records (Fig. 1) [56,57]. The latter are typically derived from conodont apatite which has the advantage that the timing of the temperature rise is well constrained by conodont biostratigraphy. The onset of this super-greenhouse began with a rapid δ^18^O negative shift indicative of a 10°C rise of ocean surface water temperatures during the course of the mass extinction implying a clear cause-and-effect link [56]. However, recent studies of δ^18^O_phosphate_ suggest that warming began at the end of the *C. yini* Zone [57,58]. This coincides with the level of the first mass extinction phase but, at the time of the losses, temperatures had not increased by more than a few degrees (although they were much higher by the time of the second extinction phase). This finding means that the δ^18^O_phosphate_ record is out of step with the δ^13^C record which begins rising early in the *C. yini* Zone. The decline of carbon isotope values during the PTMME is known globally and is widely regarded to reflect the emission of isotopically light carbon from the Siberian Traps. The slight mismatch in timing between the two isotopic systems may be because the initial carbon dioxide emissions were from volumetrically minor but isotopically very light (> −20‰) thermogenic sources in the vicinity of the Siberian Traps. These were replaced by more voluminous but isotopically heavier (−5‰) volcanic CO_2_ emissions when the main flood basalt eruptions began [57,59]. Thus, the initial emissions were sufficient to affect the δ^13^C record but not enough apparently to increase global temperature to the point where it registered in the δ^18^O record. If this is the case then it is difficult to see that there was a role for extreme temperatures in the first phase extinction, despite the palaeontological evidence noted above. However, other oxygen isotope data provide a clear link between carbon isotopic trends and extinctions (Fig. 1). Analysing calcite δ^18^O values from ostracods in the lead up to the PTMME, Gliwa and colleagues [60] showed there was a gradual decline (precisely coincident with the δ^13^C decline) beginning in the *C. yini* Zone. By the time extinction struck temperatures had risen by 10°C, sufficient to be a factor in any extinction scenario. Why the conodont and ostracod data should differ in their timing is unresolved and there is clearly a need for further studies of temperature proxies during the PTMME.

### Ocean acidification

In addition to its greenhouse gas effects, the rapid release of carbon dioxide during the PTMME is predicted to have reduced the pH of surface waters of the oceans to the point that it caused extinctions amongst marine life [61,62]. However, those who advocate ocean acidification have a problem: unlike the study of redox levels there are few reliable geochemical proxies for deriving ancient pH levels, boron and calcium isotopes being two of the few available. Furthermore, intense acidification will dissolve seafloor carbonates effectively removing their own geochemical evidence, though truncation surfaces remain [63].

Studying boron isotopes in biogenic calcite is analytically difficult and there are strong vital effects that must be accounted for. Nonetheless, it has provided a reasonable proxy for ocean pH in Cenozoic studies of planktonic foraminifers where species-specific vital effects are well constrained [64]. It is not possible to produce a single-species δ^11^B record from across the PTMME because no suitable species survived the crisis. Thus, studies have had to use multiple taxa or whole-rock carbonate samples. The results have, perhaps unsurprisingly, been inconsistent. A study of whole-rock carbonate from Oman showed no change in δ^11^B values across the Permo-Triassic boundary, but a substantial negative excursion (values drop from 14‰ to 8‰) in the early *I. isarcica* Zone [65]. In contrast, analysis of several different brachiopod taxa showed a more modest decline of δ^11^B values, from 14‰ to 11‰, beginning in the *H. changxingensis* Zone and culminating in the *I. isarcica* Zone [66]. These records show neither a close match with the extinction pulses nor with each other (Fig. 1).

Calcium isotope trends recorded in marine carbonates (δ^44^Ca_carb_) and biogenic phosphate reflect changes in the oceans’ values and they typically vary by about ± 20‰ on a million-year timescale [67]. The variations reflect the balance between input, from terrestrial weathering of silicates and carbonates, and output in the form of carbonate burial [61,67,68]. The burial fractionates the light isotope leaving the residual oceanic isotopic system heavier. In shallow marine carbonates from across the Permo-Triassic boundary δ^44^Ca_carb_ declines by −0.6‰ indicating a global decrease in carbonate formation; a trend that has been ascribed to ocean acidification [61]. The decline closely parallels the δ^13^C curve, beginning in the *C. changxingensis* Zone and culminating in the *H. parvus* Zone before gradually recovering in the succeeding zones, thus providing indirect evidence that carbon emissions were driving acidification [61]. However, numerous factors control the saturation state of carbonate in the ocean, not just pH, including temperature, pressure, and weathering fluxes. Using δ^44^Ca_carb_ trends to disentangle any unique cause is difficult. For example, a positive δ^44^Ca_carb_ excursion during the Cenomanian-Turonian oceanic anoxic event is suggested to indicate ocean acidification during this younger crisis [69]. If both positive and negative Ca isotope excursions can indicate acidification then such values have little value as a proxy. The negative δ^44^Ca_carb_ excursion during the PTMME could have been mostly driven by the reduction in carbonate burial caused by the extinction of most calcareous organisms [68].

The sedimentary record provides little support for carbonate under-saturation in the latest Permian because both calcimicrobial carbonates and oolitic beds are widespread throughout Tethys in the aftermath of the first phase of mass extinction [17]. The dramatic temperature rise across the Permo-Triassic boundary (see above) would increase the saturation state especially in the already warm equatorial oceans of Tethys. Furthermore, microbialites become widespread in South China in the immediate aftermath of the first phase of mass extinction, during the *H. changxingensis* Zone [70] at a time when the ongoing fall of δ^44^Ca values suggests the oceans were acidifying.

A novel approach to assessing pH levels was provided by a study of marcasite in the Meishan GSSP [47]. This iron sulphide phase forms in acidic conditions (pH <5.5) and it becomes abundant in Beds 26–32 at the global stratotype and shows a decline in δ^34^S_marcasite_ values to −50‰ indicating formation in open porewaters. Build-up of excess H_2_S is suggested as a cause of porewater acidification and the authors of the study note that this occurs ‘broadly within the extinction interval’ [47]. However, in detail the marcasite formation clearly postdates the first phase of extinction and, whilst porewaters may have been acidic in some locations such as Meishan, the interlude between the two phases of mass extinction coincides with the microbialite and oolite formation noted above.

More physical evidence for ocean acidification has been suggested, such as truncation surfaces in tropical carbonate successions straddling the Permo-Triassic boundary [63] but these are alternatively interpreted as exposure surfaces [71]. Perhaps the best approach to deciphering the potential role of ocean acidification in the mass extinction comes from palaeontological data. Aspects of extinction selectivity have also been used to support an acidification kill mechanism, such as the preferential loss of coarsely ornamented ammonoids relative to smooth forms [72], but other explanations are possible. Smooth ammonoid survival could simply reflect a more opportunistic life strategy that favours simple morphology with a focus on rapid reproduction rates, better suited to survival in high stress environments. The small size of many survivors (the Lilliput Effect) could also reflect adaption to acidified oceans in the aftermath of end-Permian losses [73]. Once again, other reasons for such a trend are possible: high juvenile mortality rates, opportunistic lifestyles in high stress environments and preferential loss of large taxa can all produce small-bodied fossil assemblages in an extinction's aftermath [74]. Other palaeoecological studies have noted that teratological evidence for acidification is lacking during the PTMME [75]. More broadly, it has been argued that the selectivity of the extinction was most severe for those organisms with thicker shells and that this reflected the difficulty of secreting carbonate in acidified waters [61]. However, the effects of both extinction phases were intense across a broad range of organisms, not just amongst the thick-shelled organisms, including those with thin shells (e.g. ammonoids), and those with siliceous skeletons, such as sponges and radiolarians, that are much less affected by lowered pH [2]. Furthermore, recent studies of large databases have failed to replicate the thick-shelled selectivity [53,76]. Despite numerous claims, there is currently no compelling evidence that ocean acidification had a role in the PTMME. Nonetheless, carbonate undersaturation may have been a severe stressor in northern Boreal latitudes prior to the end of the Permian. The disappearance of shelly groups at the base of the Changhsingian in Spitsbergen has been attributed to the shoaling of the lysocline [77] and this level is marked by a major, negative carbon isotope excursion and evidence for enhanced volcanism [20,78]. Ocean acidification may have been a kill mechanism in cool northern waters during an earlier, little-known, base-Changhsingian extinction event that was distinct from the PTMME 2.3 million years later.

### Siltation

Global run-off is widely regarded to have increased in step with warming during the Permo-Triassic transition [79–81] with potentially dire consequences for marine biota. Thus, Algeo and Twitchett [79] proposed a death-by-siltation model in which the greatly enhanced global influx of clay-rich sediments to marine settings caused stress for both suspension feeders and grazers and restricted photosynthesis due to increased turbidity. In support of this model, average sedimentation rates in the Early Triassic were shown to be much higher than those of the Late Permian, with an approximately seven-fold increase in terrigenous runoff rates [79]. However, calculations of rates are dependent on the timescale used; the original work [79] used a 2004 timescale but the latest version has considerably different durations for Early Triassic intervals [82]. Of particular importance, the first Triassic substage—the Griesbachian—is now considered to have a duration of 1.01 myr rather than the 0.65 myr value used by Algeo and Twitchett [79]. This considerably reduces the calculated increase of sedimentation rates from the latest Permian to the earliest Triassic (Table 1). Furthermore, increased Triassic sedimentation rates are not a global phenomenon. The extensive shelf seas on the Perigondwanan margin (South Tibet) experienced low sedimentation rates throughout the Early Triassic, whilst there was a substantially increased sediment influx into Boreal Seas (Table 1). Another problem with the siltation extinction mechanism is the lack of terrigenous run-off into Tethyan shelf seas where carbonate deposition dominates. Algeo and Twitchett [79] note that many Tethyan sections show a transition from pure carbonates to more marly rocks, but this phenomenon occurs after the PTMME and is a function of widespread deepening that resulted in the establishment of offshore marl deposition in many sections [83,84].

**Table 1.** Comparison of sedimentation rates (m/myr) during the late Permian to early Triassic interval from three different regions, using stage durations from the Geologic Time Scale 2020 (Changxingian 2.34 myr, Griesbachian 1.10 myr, Dienerian 0.62 myr, Smithian 1.78 myr). Sverdrup data from [88,89], Yangtze Platform data from [90], Perigondwanan shelf from [91] and West Australian Shelf from [92].


	Changxingian	Griesbachian	Dienerian	Smithian

Sverdrup Basin (Spath Creek)	39	59	290	185
Yangtze Platform (Yashan)	18	43	130	154
Perigondwanan shelf (Tulong)	5	0.7	4.8	1.4
West Australian Shelf (Hovea)	5	8	10	>38

The siltation model has undergone modification by the original authors (and by others in more recent publications), with the suggestion that the collapse of forest ecosystems during the terrestrial component of the PTME led to briefly enhanced erosion of freshly exposed soils [80]. The resultant ‘massive soil and rock erosion’ is said to have swamped shelf seas with an ‘enormous mud supply’ [85]. However, the problem remains that there is no evidence for the ‘enormous’ amounts of mud, especially not in the pure carbonate sections of Tethys. There are also timing problems with this modified model. Terrestrial vegetation collapse predated the first phase of marine extinction by tens to hundreds of thousands of years [86,87] making any immediate cause-and-effect link with the marine extinctions unlikely.

### Primary production

The PTMME marks a fundamental change in the composition of both phytoplankton communities and the heterotrophic radiolarians. The latter underwent a major evolutionary bottleneck: only a few species are known from the Early Triassic [93] and an Early Triassic ‘chert gap’ in oceanic sediments indicates a collapse of siliceous productivity. Diverse lines of evidence show that the productivity collapse was initiated around the Permo-Triassic boundary. Organic biomarkers indicate a shift from plankton populations dominated by eukaryotic algae in the latest Permian to communities composed of green-sulphur bacteria, sulphate-reducing bacteria, cyanobacteria, prasinophytes and acritarchs during and after the PTMME interval [94–96]. The presence of the first two bacterial groups on the list suggests the development of widespread anoxia in the shallow waters of the oceans for which there is also ample inorganic geochemical evidence (see above). Contemporary organic nitrogen isotope (δ^15^N_org_) trends also show major changes: values decline from 3‰–4‰ in the latest Permian to 0‰ in the Early Triassic [24,94,97] although there are regional variations [98]. Significant denitrification occurs in oxygen-poor waters leaving surface waters enriched in ^15^N which is reflected in the heavy δ^15^N_org_ values around the extinction level. However, once nitrate becomes a limiting nutrient (for example if there is a major decrease in the upwelling renewal of nitrate), then nitrogen-fixing, diazotrophic cyanobacteria dominate which have a characteristic δ^15^N_org_ value of ∼0‰ [99]. These changes suggest that a decline in primary productivity was a potential contributory factor in the PTMME although in many cases the excursion slightly postdates the first phase of the extinction [99].

The much smaller size of cyanobacteria-dominated planktonic communities, compared with the eukaryotic populations of the Late Permian, could also have decreased grazing by zooplankton, and prolonged the residence time of organic matter in the water column thereby increasing oxygen demand and the intensity of anoxia [2,100]. However, in detail, the transition to 0‰ δ^15^N_org_ values occurs at different levels in different sections. Shallow water platform carbonate locations in South China show the decline precisely at the level of the first phase of mass extinction [24], but the presence of a hiatus at the same level makes the precise relationship moot. Clearer evidence comes from the stratigraphically complete Meishan stratotype. Here, the δ^15^N_org_ values drop from 2.5‰ to 0‰ at the Bed 24/25 boundary [101], the level of the first phase losses. The corresponding peak of methyl hopanes (cyanobacteria biomarkers) in beds 24–37 at Meishan supports the N isotopic evidence for N fixation becoming important at this time [94]. However, some but not all more offshore sections in South China exhibit low δ^15^N_org_ values before the PTMME suggesting N fixation was already occurring in these locations before expanding greatly during the crisis [24,98]. Other regions of the world also show declining productivity prior to the extinction [20]. Therefore, whilst there was a long-term trend to less productive oceans, the role of primary production fluctuations during the mass extinction phases is not clear.

There are two diametrically opposed schools of thought on what happened to primary productivity during the PTMME: either it dramatically increased or it collapsed. The former alternative is attributed to increased influx of terrestrial nutrients in a rapidly warming and humidifying world potentially exacerbated by soil erosion following collapse of terrestrial plant communities [80,100,101] or from the enhanced influx of volcanic nutrients [102,103]. Alternatively, there was a catastrophic productivity crash because deepening of the nutricline in stratified waters and/or shutdown of upwelling starved the surface waters of nutrients [18,43,104]. In the former alternative, eutrophic conditions are not a direct cause of extinction (abundant food is not stressful for life!), but they are often linked with an expanded oxygen-minimum zone (which is). On the other hand, intense oligotrophy (nutrient starvation) is a direct stress. The small size of survivors in the aftermath of the mass extinction—the aforementioned Lilliput Effect—has led to the suggestion that the crisis was caused by the loss of larger organisms with their greater food requirements [105]. Such a stressor might be anticipated to shorten food chains as the energy transfer between feeding levels decline. But, this prediction is not supported by the fossil data. Groups at the top of the food chain, notably fish, survived the PTMME substantially better than other groups [106]. This was a crisis that simplified but did not truncate food webs [76]. As noted above, there are many other reasons why small species should proliferate in the aftermath of a mass extinction.

The role of productivity fluctuations during the PTMME remains intriguing. The radiolarians are the only group to provide direct evidence of the fortunes of plankton during the crisis. Interestingly, in south China, the radiolarian losses are considerably out of step with other groups: they suffer major losses (87% species extinction) in the basal *C. changxingensis* zone [25,107]. This is substantially before other extinctions and it also predates the carbon and oxygen isotope excursions associated with the PTMME. It is suggested that the losses may coincide with a decline in productivity [107] but the cause is unclear. Panthalassa was a major site of radiolarian chert accumulation throughout the Permian and the available data suggests losses began in this ocean, at least at the margins, before the main extinction phases. Thus, in western Canada on the eastern side of Panthalassa most radiolarian losses predate the benthic extinctions with all but simple sphaeroid-forms of radiolarians disappearing before the benthic losses (of siliceous sponges) [18]. The available C isotope stratigraphy indicates the Canadian radiolarian crisis occurs at the onset of the δ^13^C_org_ negative excursion which supports the idea that this group's crisis began before the first phase of the PTMME which occurred during the later stages of the negative excursion (Fig. 1). In contrast, the mid-southern latitude oceanic section at Arrow Rocks in New Zealand shows several radiolarian taxa (10 genera) surviving into the earliest Triassic Induan Stage [108]. The cause of the radiolarian crisis remains enigmatic because its onset predates the major environmental changes of the main mass extinction, even if the final losses are clearly linked with the development of ocean anoxia and warming [30,108,109].

### Other causes

Large-scale volcanic eruptions cause global cooling due to the emission of SO_2_ and the formation of sulphate aerosols in the atmosphere. If the gases reach the stratosphere, such effects persist for a few years-to-decades before being rained out. The scale of the Siberian Traps flood basalt eruptions was likely sufficient to achieve such short-term cooling episodes and ‘volcanic winter’ extinction scenarios became popular once the temporal link between eruptions and extinctions was established around 30 years ago [110]. However, this idea fell out of favour within a few years with the discovery of abundant and diverse evidence for rapid global warming during the extinction (see above). Nonetheless the idea still has proponents [111,112]. Signs of a short-lived, minor regression are seen around the level of the first phase of mass extinction in shallow-water sections of South China and this is overlain by microbial carbonates of the interlude interval. By attributing the sea-level fall to a brief continental glaciation, Baresel and colleagues [111] reintroduced a cooling-driven extinction model, although their assertion that ‘the build-up of volcanic CO_2_ induced a transient cool climate whose early phase saw the deposition of microbial limestone’ is highly unlikely, or else there is a typographical error in this statement. In further support, Zhang *et al.* [112] noted that there is a brief 4°C cooling episode recorded at the Penglaitan boundary section which they suggest indicates a brief volcanic winter. However, this cooling is based on a single δ^18^O_phosphate_ data point from basal Triassic strata and does not correlate with either phase of the PTMME. One would hope for more substantial evidence for cooling if volcanic winter is to be considered a viable kill mechanism for the PTMME.

Finally, Permo-Triassic boundary sections often show enrichment in trace metals, notably copper and mercury, that are likely volcanically derived, either from Siberian or continental arc magmatism which was especially intense in South China [112]. Such enrichment, especially of mercury, is a good marker for large-scale volcanism but it is also possible that it is a contributory cause of extinction. Mercury is highly toxic and may have led to direct mortality in the oceans [113] although whether levels were ever sufficient to cause extinctions is debatable.

## SUMMARY AND FUTURE RESEARCH TARGETS

Based on the evidence amassed over the past 30 years the course of the PTMME is now well documented, especially in the equatorial waters of South China. Most losses occurred in a ∼60 kyr interval straddling the Permo-Triassic boundary with the majority of extinctions occurring at the beginning and end of this interval. The first phase has traditionally been identified as the main extinction level, hence the frequent reference to an ‘end-Permian mass extinction’. Evidence from Boreal latitudes suggests the first extinction phase is also manifest in this region too although shelly benthos had already disappeared from this region at the base of the Changxingian. Further research is needed on this interval to determine if this constitutes a distinct crisis prior to the PTMME.

The Permo-Triassic extinctions were severe for virtually all groups, although nekton such as fish and conodonts (but not ammonoids) faired relatively well, indicating that the crisis was at its most intense on the seafloor. This matches with the widespread evidence for seafloor anoxia seen globally at this time, although warming was likely a contributory factor that may have been responsible for the slightly earlier onset of losses amongst radiolarians. Changes in primary productivity levels during the crisis were substantial, with bacterial groups being the main beneficiaries, but whether the crisis saw eutrophic or oligotrophic oceans is unresolved.

Voluminous carbon dioxide emissions from the Siberian Traps and the baked strata that it was emplaced within [114] are the widely accepted driver of the mass extinction. However, there is much that remains to be discovered about the causal link between the resultant warming, ocean anoxia and primary productivity changes. Current earth system modelling provides fundamentally different drivers for marine anoxia (e.g. [23] versus [48]). Ocean acidification is another stressor which again is ultimately attributable to CO_2_ emissions, but new proxies for past ocean pH or improvements in existing approaches are required to test for this kill mechanism. Finally, after decades of study, the detail and resolution of the South China fossil record is exceptionally good and most extinction scenarios rely on data from this region. Our understanding of the PTMME in other regions, especially in high latitudes, lags far behind. The extinction record from Boreal latitudes is distinctly different and it would be especially useful to expand our knowledge of this region. For example, the fossil record from north-east Siberia would provide an especially useful comparator to help understanding the global course of the world's worst crisis.
